# Inhibitory Effect of Antimicrobial Peptides Bac7(17), PAsmr5-17 and PAβN on Bacterial Growth and Biofilm Formation of Multidrug-Resistant *Acinetobacter baumannii*

**DOI:** 10.3390/microorganisms13030639

**Published:** 2025-03-11

**Authors:** Johanna Rühl-Teichner, Daniela Müller, Ivonne Stamm, Stephan Göttig, Ursula Leidner, Torsten Semmler, Christa Ewers

**Affiliations:** 1 Institute of Hygiene and Infectious Diseases of Animals, Department of Veterinary Medicine, Justus Liebig University Giessen, 35392 Giessen, Germany; johanna.ruehl@vetmed.uni-giessen.de (J.R.-T.); ursula.leidner@vetmed.uni-giessen.de (U.L.); 2 Department of Pharmaceutics and Biopharmaceutics, Philipps-Universität Marburg, 35032 Marburg, Germany; daniela.mueller@pharmazie.uni-marburg.de; 3 Vet Med Labor GmbH, 70806 Kornwestheim, Germany; ivonne-stamm@idexx.com; 4 Institute of Medical Microbiology and Infection Control, Hospital of Johann Wolfgang Goethe University, 60596 Frankfurt, Germany; goettig@med.uni-frankfurt.de; 5 NG1, Microbial Genomics, Robert Koch Institute, 13353 Berlin, Germany; semmlert@rki.de

**Keywords:** multidrug resistance, antimicrobial peptides, efflux pump inhibitor, international clones, biofilm-associated genes

## Abstract

*Acinetobacter* (*A.*) *baumannii* is a major nosocomial pathogen in human and veterinary medicine. The emergence of certain international clones (ICs), often with multidrug-resistant (MDR) phenotypes and biofilm formation (BF), facilitates its spread in clinical environments. The global rise in antimicrobial resistance demands alternative treatment strategies, such as antimicrobial peptides (AMPs). In this study, 45 human and companion animal MDR-*A. baumannii* isolates, belonging to the globally spread IC1, IC2 and IC7, were tested for antimicrobial resistance and biofilm-associated genes (BAGs) and their capacity for BF. Of these, 13 were used to test the inhibitory effect of AMPs on bacterial growth (BG) and BF through the application of a crystal violet assay. The two novel AMP variants Bac7(17) (target cell inactivation) and Pasmr5-17 (efflux pump inhibition) and the well-known AMP phenylalanine-arginine-β-naphthylamide (PAβN) were tested at concentrations of 1.95 to 1000 µg/mL. Based on whole-genome sequence data, identical patterns of BAGs were detected within the same IC. AMPs inhibited BG and BF in a dose-dependent manner. Bac7(17) and PAsmr5-17 were highly effective against BG, with growth inhibition (GI) of >99% (62.5 and 125 µg/mL, respectively). PAβN achieved only 95.7% GI at 1000 µg/mL. Similar results were obtained for BF. Differences between the ICs were found for both GI and BF when influenced by AMPs. PAsmr5-17 had hardly any inhibitory effect on the BF of IC1 isolates, but for IC2 and IC7 isolates, 31.25 µg/mL was sufficient. Our data show that the susceptibility of animal MDR-*A. baumannii* to AMPs most likely resembles that of human isolates, depending on their assignment to a particular IC. Even low concentrations of AMPs had a significant effect on BG. Therefore, AMPs represent a promising alternative in the treatment of MDR-*A. baumannii*, either as the sole therapy or in combination with antibiotics.

## 1. Introduction

*Acinetobacter* (*A*.) *baumannii* is a Gram-negative bacterium of the family *Moraxellaceae* that causes hospital- and community-associated infections such as urinary and respiratory tract infections, bacteremia, sepsis and wound infections [1,2,3]. It possesses several mechanisms of antimicrobial resistance (AMR), often leading to a multidrug-resistant (MDR) phenotype, including drug-modifying enzymes, efflux pump hyperproductivity, membrane permeability defects and target site alteration [4]. Due to its successful dissemination in different environments, *A. baumannii* belongs to the ESKAPE group, in which treatment failures due to antibiotic resistance are common [5].

In recent years, *A. baumannii* has also been recognized as a serious pathogen in animals, particularly due to its prevalence in veterinary clinics [6]. Animals often carry the same clones found in humans. To date, eleven international clones (ICs) of *A. baumannii* have been identified, each representing at least one specific sequence type (ST): IC1 (ST1), IC2 (ST2), IC3 (ST3), IC4 (ST15), IC5 (ST79), IC6 (ST78), IC7 (ST25), IC8 (ST10), IC9 (ST85), IC10 (ST158) and IC11 (ST164) [7,8,9,10]. IC1, IC2 and IC7 are of particular interest as they are responsible for the majority of nosocomial and community-acquired *A. baumannii* infections worldwide [6,11].

The ability of *A. baumannii* to form biofilms probably facilitates urinary and respiratory tract infections in humans and animals [6,12,13]. Biofilms enable growth on abiotic surfaces, such as stainless steel and polystyrene, used in medical devices including urinary catheters and endotracheal tubes [14,15,16]. MDR and the ability of biofilm formation (BF) allow *A. baumannii* to survive in the harsh hospital environment for extended periods of time. Consequently, *A. baumannii* infections in both human and veterinary medicine are difficult to control [17]. BF increases the antibiotic resistance of *A. baumannii*, and in veterinary medicine, it is a significant factor in recurrent infections, particularly urinary tract infections [18]. There is limited evidence on the correlation between the ability of BF and the AMR phenotype of *A. baumannii* isolates. Certain genes that contribute to the virulence of the pathogen are also associated with BF and are referred to as biofilm-associated genes (BAGs) [15,19,20,21,22,23].

The treatment of *A. baumannii* infections is challenging due to their intrinsic and acquired resistance to many antibiotics. In human medicine, monotherapy with minocycline can be used for mild-to-moderate infections [24]. Severe *A. baumannii* infections are usually treated with combination therapy, such as carbapenems (imipenem/meropenem) or ampicillin–sulbactam along with an aminoglycoside [25]. Colistin or tigecycline is recommended for MDR infections if no alternative antibiotics are effective [26,27,28]. In veterinary medicine, an antibiotic is selected on the basis of antimicrobial susceptibility [29]. *A. baumannii* is increasingly recognized as a serious pathogen in animals [6]. Resistance to penicillins, cephalosporins and tetracyclines complicates the treatment of *A. baumannii* infections [30,31]. More and more isolates are considered MDR or even extensively drug-resistant, which severely limits treatment options [6,24].

With the increase in MDR-*A. baumannii*, this pathogen was added to the World Health Organization’s (WHO) list of priority pathogens for the research and development of new therapeutic options in 2017 [32,33]. Antimicrobial peptides (AMPs) represent a promising approach for the treatment of *A. baumannii* infections due to their high activity and efficiency against various bacteria [34]. As part of the natural immune system of prokaryotes and eukaryotes, they fight potential pathogens or regulate the body’s own microbiome [35,36]. AMPs are 11–50-amino-acid-long, amphipathic molecules that bind to bacterial membranes and enter the bacteria by permeabilization or pore formation [37,38]. The mode of action of AMPs is not yet fully understood. However, it is known that the main mechanism of antimicrobial activity is based on a non-receptor-mediated membrane disruption. In addition, AMPs can cause cell death by inhibiting protein/DNA or cell wall synthesis or by inducing apoptosis and necrosis [39]. Many AMPs have already shown inhibitory effects on the bacterial growth (BG) and BF of certain bacterial species [40]. In this study, three AMPs with different modes of action were selected to test their efficacy against BG and BF in MDR-*A. baumannii* isolates: Bac7(17) (target cell inactivation and protein synthesis inhibition), phenylalanine-arginine-β-naphthylamide (PAβN; efflux pump inhibition) and the synthetic PAsmr analogue PAsmr5-17 (efflux pump inhibition) [41,42,43,44].

While PAβN has been extensively studied, Bac7(17) and PAsmr5-17 represent completely novel AMP variants that have not been examined before. The aim of this study was to analyze the inhibitory effect of these new AMP variants on BG and BF. The AMPs were tested for their effectiveness in human and, for the first time, animal MDR-*A. baumannii* isolates (dogs and cats). The results might offer a unique opportunity to optimize the future treatment of MDR-*A. baumannii* infections in both human and veterinary medicine.

## 2. Materials and Methods

### 2.1. Bacterial Strains and Growth Conditions

In this study, 13 *A. baumannii* isolates (Table 1) were examined, which were selected on the basis of the following criteria from a pool of in-house isolates: classified as MDR according to Magiorakos et al. (2012) [45], belonging to a specific ST/IC (ST1/IC1, ST2/IC2 or ST25/IC7), isolated from humans, dogs or cats and have the ability to produce strong biofilm. The isolates were either collected by an in-house microbiological diagnostic laboratory or provided by external veterinary and human medical diagnostic laboratories. The *A. baumannii* reference strains ATCC 19606^T^ (human, urinary tract) and ATCC 17978 (human, meningitis) were used as controls. The species identification of clinical isolates was performed with matrix-assisted laser desorption time-of-flight mass spectrometry (MALDI-TOF-MS) using the Microflex LT/SH mass spectrometer and the biotyper database (MALDI Biotyper V3.3.1.0) (both Bruker Daltonics, Bremen, Germany). Additionally, species identification was assessed using ribosomal multilocus sequence typing (rMLST) and the online tool “Identify species” offered by PubMLST (https://pubmlst.org/species-id (accessed on 10 February 2025)). All isolates were stored at −70 °C in Brain Heart Infusion Broth (Oxoid, Wesel, Germany) containing 30% glycerol.

### 2.2. Antimicrobial Susceptibility Testing (AST)

AST was carried out using the VITEK^®^2 compact automated instrument system (AST-cards GN-97 and AST-N248; bioMérieux, Nürtingen, Germany) during routine laboratory diagnostics [46]. The antibiotics tested were ampicillin (AMP), amoxicillin–clavulanic acid (AMC), piperacillin (PIP), piperacillin–tazobactam (TZP), cephalexin (CEX), cefotaxime (CTX), ceftazidime (CAZ), imipenem (IPM), meropenem (MEM), amikacin (AMK), gentamicin (GEN), tobramycin (TOB), ciprofloxacin (CIP), enrofloxacin (ENR), marbofloxacin (MAR), tetracycline (TET), chloramphenicol (CHL), colistin (COL) and trimethoprim/sulfamethoxazole (SXT). Since no minimal inhibitory concentrations (MICs) were defined by CLSI documents VET01S and VET09 for *Acinetobacter* spp., MICs were interpreted according to human-derived breakpoints from CLSI document M100 for *Acinetobacter* spp. [47] with the exception of AMC and CHL (breakpoints for *Enterobacterales*, CLSI), AMP, CEX and COL (breakpoints for *Enterobacterales*, EUCAST [48]) and ENR and MAR (breakpoints for ciprofloxacin, *Acinetobacter* spp., CLSI). Intrinsic resistance was assumed in accordance with the CLSI guideline definitions [47]. *Escherichia coli* ATCC 25922 and *Pseudomonas aeruginosa* ATCC 27853 were used as quality-control reference strains for AST performed on the VITEK^®^ 2 compact automated instrument system as recommended by the manufacturer.

### 2.3. Whole-Genome Sequencing

The DNA for whole-genome sequencing (WGS) was extracted by using the “Master Pure™ DNA Purification Kit” (Biozym Scientific GmbH, Hessisch Oldendorf, Germany). Sequencing libraries were prepared using the Nextera XT Library Preparation Kit (Illumina GmbH, Munich, Germany) for a 250 bp paired-end sequencing run on an Illumina MiSeq sequencer (Illumina Inc., San Diego, CA, USA) with a minimum coverage of 100-fold. FASTQ files were quality-trimmed before they were assembled de novo and annotated using SPAdes v.3.15.5 (https://github.com/ablab/spades/releases/tag/v3.15.5 (accessed on 10 February 2025)) and RAST v.2.0 (http://rast.nmpdr.org/ (accessed on 10 February 2025)).

### 2.4. Multilocus Sequence Typing, Antibiotic Resistance Genes and Biofilm-Associated Genes

WGS data were used to determine AMR genes and BAGs using the online tools “ResFinder 4.4.2”, provided by the Center for Genomic Epidemiology (CGE) [49,50,51], “single genome analysis”, provided by BacWGSTdb 2.0 [52,53,54], and “MyDBFinder”, provided by CGE (https://cge.food.dtu.dk/services/MyDbFinder/ (accessed on 10 February 2025)), with a custom in-house database containing BAGs. These BAGs include *csuA/B/C/D/E* (chaperone–usher pilus Csu), *ompA* (outer membrane protein A), *bap* (biofilm-associated protein), *blp1* and *blp2* (Bap-like proteins), AbaIR (quorum-sensing system, consisting of *abaI* (acyl-homoserine lactone synthase AbaI) and *abaR* (DNA-binding HTH domain-containing protein AbaR), PNAG (polysaccharide poly-β-(1,6)-N-acetylglucosamine), which is encoded by the gene cluster *pgaABCD*, and *bfmRS* (two-component signal transduction, consisting of *bfmR* (response regulator BfmR) and *bfmS* (sensor kinase BfmS)) [16,19,20,21,22,23].

A threshold of 90% for both nucleotide identity and length coverage was set for the prediction of AMR genes and BAGs. Ridom v10.0.5 (Ridom GmbH, Münster, Germany) and Geneious 8.1.9 (Biomatters Ltd., Auckland, New Zealand) and subsequent nucleotide BLAST of the National Center of Biotechnology Information (https://blast.ncbi.nlm.nih.gov/Blast.cgi (accessed on 10 February 2025)) were used to identify the exact location (chromosome or plasmid) of the *bla*_OXA_ genes. Multilocus sequence types were determined using CGE’s “MLST 2.0” (Pasteur scheme) online tool (https://cge.food.dtu.dk/services/MLST/ accessed on 10 February 2025) [55,56].

### 2.5. Minimum Inhibitory Concentration (MIC) Assay

The AMPs Bac7(17) and PAsmr5-17 were provided by Dr. Daniela Müller. PAβN was purchased from Sigma-Aldrich/Merck (Darmstadt, Gemany). Stock solutions of AMPs were prepared at a concentration of 1 mg/mL in distilled water, and a concentration range of 1.95 μg/mL to 1000 μg/mL was generated by a 1:2 dilution series in distilled water. MIC testing was performed by a microbroth dilution assay [42,57]. For this purpose, 20 μL of each AMP per well was added to a 96-well microtiter plate (MTP). The plates were stored at −20 °C until further use. An overnight culture of each isolate was inoculated into Mueller-Hinton Broth (MHB; Merck KGaA, Darmstadt, Germany) and incubated for 20-24 h at 37 °C on a shaker at 130 rpm. A 1:1000 dilution of each overnight culture was prepared with fresh MHB. The bacterial suspension was added to the prepared 96-well MTPs up to a final volume of 120 µL in each well. The plates were covered with a lid and incubated at 37 °C on a shaker at 130 rpm for an additional 24 h. Growth inhibition was determined photometrically by using the Multiskan FC (Thermo Fisher Scientific, Waltham, MA, USA) at OD_595_. All assays were performed in triplicate, and the results were averaged. Percentage growth inhibition was determined using the following formula:

Growth inhibition (%) = 1 − ((OD595_inhibited_ − OD595_blank_)/(OD595_uninhibited_ − OD595_blank_)).

The optical density is measured at 595 nm (OD_595_). OD595_blank_ indicates sterile medium without bacteria, OD595_uninhibited_ indicates uninhibited BG without test substance after 24 h, and OD595_inhibited_ indicates BG inhibited by the respective substance after 24 h.

The MIC describes the lowest concentration at which no BG can be detected. In addition to the complete inhibition of BG (100%), called MIC100, other partial inhibitions such as MIC90 or MIC50 can also be specified, at which 90% or 50% growth inhibition can be determined.

### 2.6. Biofilm Assay

A crystal violet assay, slightly modified from O’Toole et al. (2011) [58], was used to quantify biofilm production. Three to five colonies of *A. baumannii* isolates were collected from blood agar plates (blood agar base from Merck Chemicals, GmbH, Darmstadt, Germany, supplemented with 5% sheep blood), resuspended in 3 mL MHB and incubated for 18 h at 37 °C on a shaker at 130 rpm. The bacterial suspension was then adjusted to an OD_600_ of 0.05. To determine the influence of AMPs on BF, 20 μL of each AMP per well with a concentration ranging from 1.95 μg/mL to 1000 μg/mL was added to a 96-well MTP. The bacterial suspension was added to the prepared 96-well MTPs up to a final volume of 120 µL per well. Biofilms were cultured in 96-well MTPs (F-Profile, Rotilabo^®^ from Carl Roth, Karlsruhe, Germany) sealed with Breathe-Easy^®^ sealing membrane (Diversified Biotech) for 24 h at 37 °C. After incubation, supernatants were removed, and each well was washed three times with distilled water. The staining of the bacterial biofilms was performed with a 0.1% (*w*/*v*) crystal violet solution (Merck KGaA, Darmstadt, Germany). After three washing steps with distilled water, the bound crystal violet was eluted by adding a solution of 80% ethanol and 20% acetone and shaking the plates for 45 min at 150 rpm. The amount of eluted crystal violet was used as a surrogate for the produced mass of biofilm. The concentration was measured photometrically using the Multiskan FC (Thermo Fisher Scientific) at OD_570_ and OD_595_. The *A. baumannii* reference strains ATCC 19606^T^ and ATCC 17978 were used as positive and negative controls, respectively [59,60]. All assays were performed in triplicate, and the results were averaged. For further statistical analysis, the OD cut-off value (ODc) was determined, defined as three standard deviations above the mean OD of the negative control. The results were classified and categorized according to Stepanovic et al. [61] as shown below.

- Non-biofilm producer: OD ≤ ODc
- Weak biofilm producer: ODc < OD ≤ 2× ODc
- Moderate biofilm producer: 2× ODc < OD ≤ 4× ODc
- Strong biofilm producer: 4× ODc < OD

### 2.7. Statistical Analysis

SPSS Statistics version 27 software (IBM, Armonk, NY, USA) was used for statistical analyses. All statistical tests were two-sided, and a *p*-value of <0.05 was considered statistically significant. A two-factor analysis of variance (ANOVA) was used to conduct multiple comparisons of the effects of AMPs on BG and BF and between the influence of the AMPs and the clonal lineage of the isolates.

## 3. Results

### 3.1. Bacterial Strains

The isolates included in this study were selected on the basis of various criteria that were initially determined for a pool of isolates. They should belong to the international clones/sequence types (ICs/STs) IC1/ST1, IC2/ST2 or IC7/ST25, originate from humans, dogs or cats, exhibit an MDR phenotype and have the ability to form strong biofilms. Five isolates from each ST/IC were tested. At least one isolate from each ST was of human origin. Based on the defined selection criteria, only three test isolates could be identified for IC7/ST25 (Table 1).

### 3.2. Antimicrobial Susceptibility Testing and AMR Genes

All *A. baumannii* isolates were resistant to all tested penicillins, carbapenems and quinolones. Six isolates showed intermediate susceptibility to CTX and susceptibility to CAZ, AMK and TOB. None of the isolates were resistant to COL (Table 2). All isolates were classified as MDR (i.e., resistant to three or more classes of antibiotics) as defined in Magiorakos et al. (2012) [45].

Among the AMR genes, the intrinsic cephalosporinase gene *bla*_ADC-25_ was found in all isolates. The ESBL gene *bla*_PER-7_ was only present in the human isolate IHIT35349, while the broad-spectrum β-lactamase gene *bla*_TEM-1D_ was detected in four isolates of IC1. With regard to intrinsic oxacillinases, all *A. baumannii* isolates carried one *bla*_OXA-51-like_ gene (*bla*_OXA-69_, *bla*_OXA-66_ and *bla*_OXA-64_), which was consistent with their assignment to IC1, IC2 and IC7. The acquired carbapenemase genes *bla*_OXA-23_ (n = 9) and *bla*_OXA-58_ (n = 4) were also found in the isolates. In addition, as shown in Table 2, nine different aminoglycoside genes could be identified: *aadA1* only in IC1, *aac(3)-IIa* and *aac(6′)-Ian* only in IC7, *aph(3′)-Ib* and *aph(6)-Id* only in IC2 and IC7, and *aac(3)-Ia* only in IC1 and IC2. Of the sulfonamides, *sul2* was only found in IC2 and IC7, while *sul1* was detected in eleven isolates. Among the AMR genes, which are responsible for resistance to phenicols, *ABUW_0982* was identified in all isolates and the *catA1* and *craA* genes in five isolates. All isolates revealed mutations in *gyrA* (S80L) and *parC* (S84L, A250T), which are associated with fluoroquinolone resistance. Different tetracycline resistance genes were identified in IC1 (*tet*(A)) and IC2/IC7 isolates (*tet*(B)). Detailed results of antimicrobial susceptibility testing (AST) data, AMR genes and chromosomal mutations conferring AMR at the isolate level are provided in Supplementary Table S1.

### 3.3. Minimum Inhibitory Concentration (MIC) of AMPs

**Figure 1:**
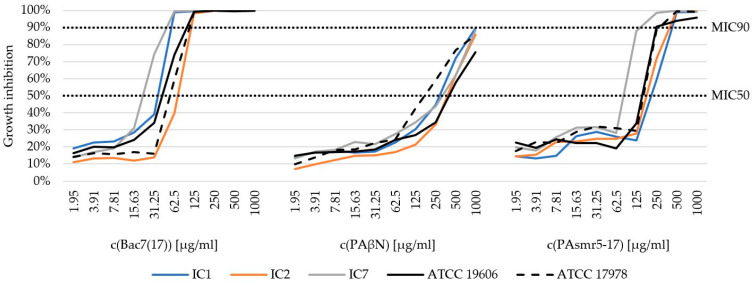
Average growth inhibition [%] by the AMPs Bac7(17), PAβN and PAsmr5-17 of the 13 MDR-*A. baumannii* isolates separated into their clonal linages: IC1 (blue), IC2 (orange) and IC7 (grey). *A. baumannii* reference strains ATCC 19606^T^ and ATCC 17978 were used as controls. MIC90: 90% growth inhibition. MIC50: 50% growth inhibition. A detailed graphical representation of individual isolates is provided in Supplementary Figure S1.

Due to the absence of breakpoints, we determined the MIC50 and MIC90 values of the three AMPs (Table 3). In general, BG was increasingly diminished with increasing concentrations of AMPs. Figure 1 shows the average percentage growth inhibition of the respective IC groups after treatment with the different AMPs. The MIC50 and MIC90 values indicate growth inhibition of 50% and 90%, respectively. A detailed graphical representation of individual isolates is provided in Supplementary Figure S1.

Bac7(17) had the strongest inhibitory effect on BG among all AMPs tested. The first MIC90 was observed at 62.5 µg/mL. Significant differences were found between Bac7(17) and PAβN at MIC50 and MIC90. While the MIC50 for Bac7(17) was already reached at 31.35 µg/mL, a concentration of 250/500 µg/mL was required for PAβN (*p* < 0.001). This was also the case for MIC90, which was only achieved for PAβN at the highest concentration of 1000 µg/mL in five isolates or, in some cases, not at all (*p* < 0.001). For PAsmr5-17, the first MIC90 was observed at a concentration of 125 µg/mL and MIC50 at an averaged concentration of 250 µg/mL. In addition, differences between the individual ICs could be observed. For Bac7(17), MIC90 was achieved at 62.5 µg/mL in the *A. baumannii* isolates assigned to IC1 and IC7. For IC2, however, MIC90 was reached at 125 µg/mL (IC1/IC7 vs. IC2: *p* < 0.001). Similar results were also obtained for PAsmr5-17. A significant difference could be observed between IC7 and IC1/IC2. For IC7, MIC90 was achieved at 125/250 µg/mL. For IC1 and IC2, on the other hand, this concentration was sufficient to achieve MIC50. The MIC90 of these clonal lineages was reached at 250/500 µg/mL (IC1 vs. IC7: *p* = 0.043; IC2 vs. IC7: *p* = 0.047).

### 3.4. Biofilm Formation (BF) and Biofilm-Associated Genes (BAGs)

**Figure 2:**
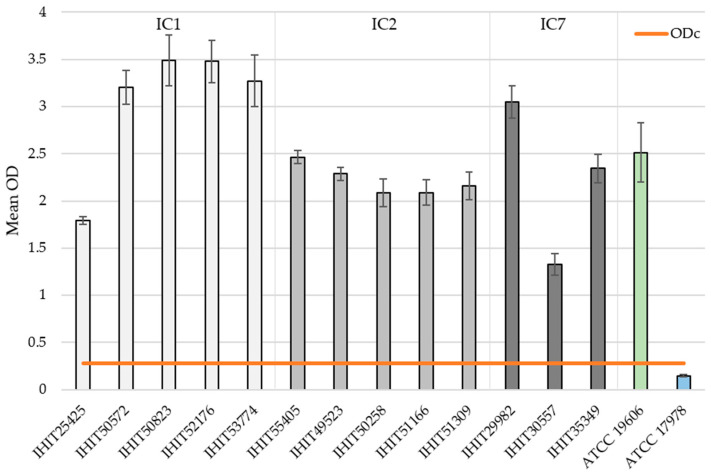
Mean OD values of BF without the influence of AMPs of the 13 *A. baumannii* isolates examined in this study sorted by their IC. Reference strains ATCC 19606^T^ (green bar) and ATCC 17978 (blue bar) were used as positive and negative controls, respectively. The orange line indicates the cut-off value (ODc) defined as three standard deviations above the mean OD of the negative control (ATCC 17978).

The biofilm-forming capacity of each isolate is summarized in Figure 2. The mean OD values for the reference strains ATCC 19606^T^ (positive control) and ATCC 17978 (negative control) were 2.514 ± 0.316 and 0.242 ± 0.011, respectively. The mean OD values for the clinical isolates ranged from 1.325 ± 0.114 (IHIT30557) to 3.491 ± 0.269 (IHIT29982). All isolates were classified into four groups based on their BF [61]. For this purpose, the cut-off value (ODc) was determined, which is defined as three standard deviations above the mean OD of the negative control. The determined ODc is 0.275. This results in the classification shown below.

- Non-biofilm producer: OD ≤ 0.275
- Weak biofilm producer: 0.275 < OD ≤ 0.550
- Moderate biofilm producer: 0.550 < OD ≤ 1.100
- Strong biofilm producer: 1.100 < OD

The classification into the corresponding groups of biofilm producers of all *A. baumannii* isolates is shown in Table 4.

Differences in the degree of biofilm formation were observed within each IC group. For example, the strains belonging to the clonal lineage IC1 show the strongest BF (Ø OD value = 3.339 ± 0.157) with the exception of isolate IHIT25425. Here, only an OD value of 1.793 ± 0.042 could be determined, which represents only 54% of the average OD value of the remaining IC1 isolates. In contrast, IC2 isolates showed a consistent result with an average biofilm OD value of 2.217 ± 0.143 (range from 2.086 to 2.463). In contrast, IC7 isolates did not present consistent BF. As mentioned above, IHIT30557 showed the lowest BF of all isolates with an OD value of 1.325 ± 0.114, followed by IHIT35349 with 2.344 ± 0.150 and IHIT29982 with 3.047 ± 0.173.

Regarding the BAGs, IC2 isolates harbored all 18 genes (Table 5). While IC1 and IC7 showed almost the same gene pattern, 3 of the 18 BAGs are missing, namely, *bap*, *blp1* and *ompA*. However, BAG profiles differed among IC1 isolates. The *A. baumannii* isolate IHIT50572 lacked the BAG *csuB*, and IHIT53774 lacked *csuA*.

### 3.5. Influence of AMPs on Biofilm Formation (BF)

**Figure 3:**
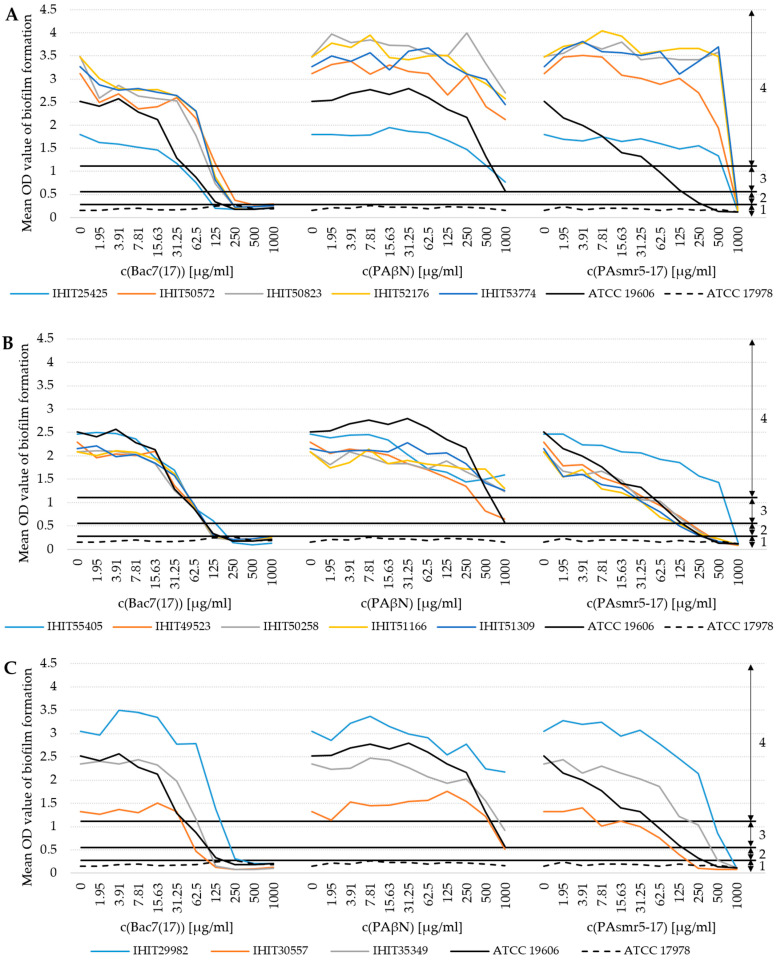
Effect of the AMPs Bac7(17), PAβN and PAsmr5-17 on the BF of the 13 MDR-*A. baumannii* isolates separated by their clonal linages: (**A**) IC1 (n = 5 isolates), (**B**) IC2 (n = 5 isolates) and (**C**) IC7 (n = 3 isolates). Reference strains ATCC 19606^T^ and ATCC 17978 were used as positive and negative controls, respectively. Areas of biofilm production: 1 = non-biofilm; 2 = weak; 3 = moderate; 4 = strong.

Of the three AMPs tested, Bac7(17) showed the strongest and PAβN the lowest inhibitory effect on BF (Figure 3). At a concentration of 62.5 µg/mL of Bac7(17), 6 of the 13 strong *A. baumannii* biofilm producers achieved moderate BF. At 125 µg/mL, only two isolates were still categorized as strong biofilm producers. At 250 µg/mL, only two isolates showed weak biofilm formation. The remaining isolates were classified as non-biofilm producers. In contrast, PAβN had hardly any inhibitory effect on BF at the same concentrations. At 500 µg/mL, one isolate could be classified as a moderate biofilm producer. At 1000 µg/mL, three isolates showed moderate biofilm formation and only one isolate showed weak biofilm formation. Unlike Bac7(17), the AMP PAsmr5-17 did not show a consistent effect on BF. There was no effect on the BF of IC1 isolates and isolate IHIT55405 (IC2). At 250 µg/mL, the first non-biofilm producer (IHIT30557) and four weak biofilm producers (all IC2, except IHIT55405) were determined. At the highest concentration, all isolates could be classified as non-biofilm producers. Despite the overall low effect of PAβN, we detected a significant difference in BF between IC1 and IC2 with an averaged OD difference of 1.331 (*p* = 0.047). Regarding PAsmr5-17, differences between IC1 and IC2 already appeared at the lowest concentration of 1.95 µg/mL, with an averaged OD difference of 1.411 (*p* = 0.050), and 3.91 µg/mL, with an averaged OD difference of 1.679 (*p* = 0.057). At 500 µg/mL, significant differences were found between IC1 and IC2 (averaged OD difference: 2.382, *p* = 0.015) as well as IC1 and IC7 (averaged OD difference: 2.401, *p* = 0.016).

## 4. Discussion

The increasing global prevalence of MDR-*A. baumannii* has prompted researchers to investigate the mechanisms of AMR in this pathogen and to develop alternative treatment strategies, both in the human and veterinary domains.

In this study, the antibacterial effect of three AMPs with different modes of action was tested to determine their potential in inhibiting BG and BF. These three AMPs are PAβN and two novel and previously untested variants Bac7(17) and PAsmr5-17. PAβN is a competitive broad-spectrum efflux pump inhibitor (EPI). This effect is particularly beneficial as the efflux pumps of bacterial cells serve as resistance mechanisms against antibiotics. Some antibiotics bind as a substrate to the efflux pump binding site and are thus transported out of the cell. Consequently, these antibiotics have no effect on the pathogen. By inhibiting these pumps, these antibiotics can no longer be transported out of the cell, resulting in a potentiation of antibiotic activity [43,62]. Bac7(17) is a proline-rich peptide derived from Bactenecin, a cathelicidine from bovine neutrophils. It has been shown to be non-toxic to mammalian cells, even at concentrations far above those effective against bacteria. Bac7(17) inactivates bacterial cells by membrane damage via depolarization, permeabilization and the subsequent inhibition of protein synthesis by binding to ribosomes [37,42]. PAsmr5-17 is a synthetic peptide-based EPI, as it contains residues centered on the TM4-TM4 binding interface found in *P. aeruginosa* small multidrug resistance (SMR) efflux protein [44]. Our study provides a comprehensive analysis of MDR-*A. baumannii* isolates from humans and animals. The isolates were assigned to either IC1 (ST1), IC2 (ST2) or IC7 (ST25). These ICs/STs are predominantly found in humans but have also often been described in *A. baumannii* infections in veterinary medicine [6,63,64,65].

The effect of AMPs on BG was determined by growth inhibition tests. Here, Bac7(17) showed the strongest influence on BG with an MIC90 of 62.5 µg/mL, followed by PAsmr5-17, revealing an MIC90 of 125 µg/mL. PAβN did not reach MIC90 and reached a maximum growth inhibition of 87.9%. These results suggest a major effect of the mode of action of the different AMPs. While PAβN as an EPI prevents the removal of metabolic products from the cell, Bac7(17) destroys the bacterial cell and inhibits protein synthesis, which leads to cell death [66]. MIC tests recently performed with pure bactenicin, which is the original source of Bac7(17), revealed an MIC95 in the species *Desulfovibrio vulgaris* at a concentration of 62.5 µg/mL, which is comparable to what we observed for *A. baumannii* and Bac7(17) [57].

Furthermore, we observed differences between *A. baumannii* clonal lineages and the effect of AMPs on BG. While 62.5 µg/mL of Bac7(17) was sufficient to achieve an MIC90 in IC1 and IC7 isolates, 125 µg/mL was required for IC2 isolates, regardless of the host of origin. A stronger effect of PAsmr5-17 was observed on IC7 isolates (MIC90 at 125 µg/mL) than on IC1 and IC2 isolates (MIC90 at 250/500 µg/mL). Overall, IC2 isolates showed the highest resistance to the AMPs, followed by IC1 and IC7 isolates.

As with BG, there was an inhibitory effect of AMPs on the BF of MDR *A. baumannii* isolates. However, it was expected that a complete inhibition of BG would result in a complete reduction in biofilm production. Therefore, we used the same isolates and applied the same growth conditions (medium, incubation time, temperature) to allow a direct comparison of MICs and data from the biofilm assay. Indeed, there was a clear correlation between growth inhibition and reduction in BF for each isolate. In addition, both Bac7(17) and PAsmr5-17 were able to reduce the biofilm-forming capacity in IC1 and IC7 isolates from the classification “strong” to “non-biofilm producer”. This finding is conclusive in that different patterns of BAGs are found in the respective ICs. IC2 isolates had all 18 of the tested BAGs, while IC1 and IC7 isolates showed an almost identical gene pattern but lacked the three BAGs *bap*, *blp1* and *ompA*. Loehfelm et al. (2008) could show that *bap* is responsible for the thickness and volume of the biofilm, among other things [67]. The weaker biofilm in IC1 and IC7 might be due to the absence of *bap* and two other BAGs, namely, *blp1* and *ompA*. Nevertheless, due to the small number of isolates tested here, further studies are required to validate and extend our results.

A lower concentration was required for Bac7(17) (125/250 µg/mL) than for PAsmr5-17 (500/1000 µg/mL), regardless of whether the isolates originated from humans or animals. A comparison with the MIC testing of AMPs shows that a higher concentration of AMPs is necessary to reduce the biofilm than to inhibit BG. This further supports the assumption that the biofilm acts as a self-protection mechanism by forming a barrier against stress caused by external influences [68]. Although PAβN resulted in biofilm reduction, the maximum reduction achieved with this AMP corresponded to “moderate biofilm producer” at the highest concentration. The minor effect of PAβN on biofilm production is probably related to its mode of action as an EPI. There are several studies on BF and efflux pumps. For example, He et al. (2015) reported that BF by clinical isolates of *A. baumannii* was associated with an overexpression of the AdeFGH efflux pump [69]. Furthermore, Richmond et al. (2016), examined the ability of different strains of *A. baumannii adeB* knockout mutants to form biofilms and showed that the knockout of *adeB* of the *adeABC* efflux pump resulted in a significant reduction in BF [70]. Our results showed that a complete inhibition of biofilm formation was not possible with PAβN. A similar result was also found by Chen et al. (2020). They achieved a 30% inhibition of the biofilm with 100 µg/mL of PAβN [71].

In summary, we showed that the novel AMPs Bac7(17) and Pasmr5-17 have a major effect on the BG and BF of MDR-*A. baumannii* isolates regardless of whether they originate from humans or animals. To the authors’ knowledge, the inhibitory effect of AMPs on the BF of MDR-*A. baumannii* isolates from companion animals was demonstrated here for the first time. Moreover, it seems that the efficacy of an AMP could be dependent on its mode of action. MDR-*A. baumannii* infections represent an increasing threat to public health. Our data are highly encouraging for future research on AMPs as alternative treatment options for infections in humans and companion animals, either as the sole medical therapy or in combination with antibiotics. Moreover, the development and evaluation of novel AMPs or AMP variants should be intensified in order to overcome the issue of limited treatment options for MDR infections.

## 5. Conclusions

AMPs are regarded as potential alternatives to antibiotics, which are becoming increasingly ineffective due to the increasing occurrence of MDR bacteria. However, due to their different mechanisms of action, AMPs cannot directly replace antibiotics. Minor changes to a peptide can affect its mode of action. It is therefore important to develop and test new AMP variants such as Bac7(17) and PAsmr7-15 and compare them with a well-researched AMP like PAβN. Our results show that low concentrations of Bac7(17) and PAsmr7-15 can significantly affect the BG and BF of MDR-*A. baumannii* isolates from humans and animals. While Bac7(17) and PAsmr5-17 were able to significantly reduce BF, PAβN showed limited efficacy. We were also able to show that AMPs with the same mode of action do not necessarily have the same effect. The differences between the individual ICs indicate that consistent results within the same species cannot be expected. This information may be of great interest for future therapies. It shows that the successful treatment of both non-MDR- and MDR-*A. baumannii* infections may depend on which IC or ST the species belongs to. These results emphasize the need for further research to evaluate the potential of AMPs as new therapeutic options to combat MDR-*A. baumannii* infections to further explore strain-specific effects. With the growing threat of MDR bacteria, it is critical to develop and explore innovative approaches to treat these infections.

## Figures and Tables

**Table 1:** Characteristics of the 13 MDR-*A. baumannii* isolates examined in this study sorted by IC.

Isolate ID	Original Name	Host	Source	Date of Isolation	Country	IC	ST^Pa^	Acquired OXA ^1^	Intrinsic OXA
IHIT25425	22/09	Human	Trachea	30.06.2009	DE	1	1	23^PL^	69
IHIT50572	VB939187.1	Cat	Urine	22.12.2022	DE	1	1	58^PL^	69
IHIT50823	VB949552.2	Dog	Wound	16.01.2023	DE	1	1	58^PL^	69
IHIT52176	VB948535	Dog	Wound	20.04.2023	DE	1	1	58^PL^	69
IHIT53774	VB952338	Dog	Urine	03.02.2023	DE	1	1	58^PL^	69
IHIT55405	34/15-1	Human	Unknown	21.12.2023	BG	2	2	23^PL^	66
IHIT49523	2214/22-1	Cat	Abscess eye	12.09.2022	DE	2	2	23^PL^	66
IHIT50258	2814/22	Dog	CVC	23.11.2022	DE	2	2	23^PL^	66
IHIT51166	237/23	Dog	Skin suture	08.02.2023	DE	2	2	23^PL^	66
IHIT51309	314/23-1	Dog	Wound	22.02.2023	DE	2	2	23^PL^	66
IHIT29982	BF135647	Dog	Urine	27.07.2015	FR	7	25	23^CH^	64
IHIT30557	BF136700	Dog	Urine	09.12.2015	FR	7	25	23^CH^	64
IHIT35349	SG1998	Human	GIT	06.07.2014	DE	7	25	23^CH^	64

^1^ Location of *bla*_OXA_ gene on plasmid (PL) or chromosome (CH). Abbreviations: CVC—Central venous catheter; GIT—Gastrointestinal tract; DE—Germany; BG—Bulgaria; FR—France; IC—international clone; ST^Pa^—multilocus sequence type according to the Pasteur scheme.

**Table 2:** AMR phenotypes and genes of 13 MDR-*A. baumannii* isolates examined in this study.

Antibiotic Class	Phenotypic Resistance			Genotypic Resistance	
	Antibiotic	Abbr.	Resistant Isolates (n)	AMR Genes	Positive Isolates (n)
β-Lactams	Ampicillin *	AMP	13		
	Amoxicillin–Clavulanate *	AMC	13	*bla*_ADC-like_	13
	Cefalexin *	CEX	13	*bla*_OXA-23_	9
	Cefotaxime	CTX	13	*bla*_OXA-58_	4
	Ceftazidime	CAZ	7	*bla*_OXA-64_	3
	Cefepime	FEP	13	*bla*_OXA-66_	5
	Imipenem	IPM	13	*bla*_OXA-69_	5
	Meropenem	MEM	13	*bla*_PER-7_	1
	Piperacillin	PIP	13	*bla*_TEM-1D_	4
	Piperacillin–Tazobactam	TZP	13		
Aminoglycosides				*aadA1*	6
				*aac(3)-Ia*	9
				*aac(3)-IIa*	3
	Gentamicin	GEN	13	*aac(6′)-Ian*	3
	Amikacin	AMK	5	*aac(6′)-Ip*	3
	Tobramycin	TOB	5	*aph(3′)-Ia*	4
				*aph(3′)-VIa*	2
				*aph(3″)-Ib*	7
				*aph(6)-Id*	7
Phenicols				*ABUW_0982*	13
	Chloramphenicol *	CHL	13	*catA1*	5
				*craA*	5
Sulfonamides/ Trimethoprims	Trimethoprim–Sulfamethoxazole	SXT	13	*sul1*	11
				*sul2*	7
Tetracyclines	Tetracycline	TET	13	*tet*(A)	5
				*tet*(B)	7
Fluoroquinolones	Ciprofloxacin	CIP	13	none **	−
	Enrofloxacin	ENR	13		
	Marbofloxacin	MAR	13		
Polymyxine	Colistin	COL	0	none	−

* Intrinsic resistance according to CLSI [47]; ** ENR/MAR-resistant isolates that revealed mutations in GyrA (S80L) (100%) and/or ParC (S84L, A250T) (100%). For *Acinetobacter* spp. isolates for which veterinary clinical breakpoints were not available, human clinical breakpoints from CLSI document M100 [37] were used where possible. For ENR/MAR, breakpoints for ciprofloxacin (CLSI) [47], for AMC/CPD/CHL, breakpoints for *Enterobacterales* (CLSI) [47], and for AMP/CEX/COL, breakpoints for *Enterobacterales* (EUCAST) [48] were used. Accession Nos. of AMR gene sequences are provided in Supplementary Table S1.

**Table 3:** MIC [µg/mL] at 50% (MIC50) and 90% (MIC90) growth inhibition for the 13 MDR-*A. baumannii* isolates and 2 reference strains for the three AMPs sorted by their IC.

		Bac7(17)		PAβN		PAsmr5-17	
Isolate	IC	MIC50	MIC90	MIC50	MIC90	MIC50	MIC90
IHIT25425	1	* 31.25	62.5	250	>1000	* 125	250
IHIT50572	1	31.25	62.5	250	1000	250	500
IHIT50823	1	31.25	62.5	500	1000	250	500
IHIT52176	1	31.25	62.5	250	1000	250	500
IHIT53774	1	* 31.25	62.5	500	>1000	250	500
IHIT55405	2	62.5	125	500	>1000	250	500
IHIT49523	2	* 62.5	125	500	>1000	250	500
IHIT50258	2	* 62.5	125	250	>1000	* 125	250
IHIT51166	2	* 62.5	125	500	1000	250	500
IHIT51309	2	* 62.5	125	500	>1000	250	500
IHIT29982	7	31.25	62.5	500	>1000	* 62.5	125
IHIT30557	7	15.63	62.5	250	1000	62.5	125
IHIT35349	7	31.25	62.5	500	>1000	125	250
ATCC 19606^T^	-	62.5	125	500	>1000	* 125	250
ATCC 17978	-	62.5	125	250	>1000	* 125	500

* These values correspond to the next lowest concentration of MIC90, as MIC50 could not be determined. MIC90: 90% growth inhibition. MIC50: 50% growth inhibition.

**Table 4:** Mean OD of biofilm formation (BF), standard deviation (SD) and corresponding classification into four different groups of biofilm producers sorted by IC.

Isolate	IC	BF	SD	Classification
IHIT25425	1	1.792	±0.042	strong
IHIT50572	1	3.113	±0.179	strong
IHIT50823	1	3.491	±0.269	strong
IHIT52176	1	3.480	±0.224	strong
IHIT53774	1	3.273	±0.275	strong
IHIT55405	2	2.463	±0.069	strong
IHIT49523	2	2.289	±0.070	strong
IHIT50258	2	2.086	±0.150	strong
IHIT51166	2	2.090	±0.134	strong
IHIT51309	2	2.158	±0.148	strong
IHIT29982	7	3.047	±0.173	strong
IHIT30557	7	1.325	±0.114	strong
IHIT35349	7	2.344	±0.150	strong
ATCC 19606^T^	-	2.514	±0.316	strong
ATCC 17978	-	0.242	±0.011	none

**Table 5:** Distribution of biofilm-associated genes among 13 clinical *A. baumannii* isolates and reference strains ATCC 19606^T^ and ATCC 17978.

Isolate	IC	Biofilm-Associated Genes (BAGs)																	
		*abaI*	*abaR*	*bap*	*blp1*	*blp2*	*bfmR*	*bfmS*	*csuA*	*csuA/B*	*csuB*	*csuC*	*csuD*	*csuE*	*ompA*	*pgaA*	*pgaB*	*pgaC*	*pgaD*
IHIT25425	1																		
IHIT50572	1																		
IHIT50823	1																		
IHIT52176	1																		
IHIT53774	1																		
IHIT55405	2																		
IHIT49523	2																		
IHIT50258	2																		
IHIT51166	2																		
IHIT51309	2																		
IHIT29982	7																		
IHIT30557	7																		
IHIT35349	7																		
ATCC 19606^T^	nt																		
ATCC 17978	nt																		

Gray-shaded cells indicate presence and white cells indicate absence of genes. nt: unclustered. BAGs were identified by screening WGS data of *A. baumannii* isolates against BAG sequences of *A. baumannii* reference strains ATCC 17978 (Accession no. CP018664.1) and AYE (only for *blp1* and *blp2*; Accession no. CU459141.1).

## Data Availability

The original contributions presented in this study are included in the article/Supplementary Material. Further inquiries can be directed to the corresponding author.

## Supplementary Materials

The following supporting information can be downloaded at https://www.mdpi.com/article/10.3390/microorganisms13030639/s1, Figure S1: Detailed graphical representation of MIC assay results of each of the 13 MDR-*A. baumannii* isolates; Table S1: Detailed list with antimicrobial resistance genes, antimicrobial susceptibility and biofilm-associated genes of the 13 MDR-*A. baumannii* isolates.
