# Supplementary material for: Inhibitory Effect of Antimicrobial Peptides Bac7(17), PAsmr5-17 and PAβN on Bacterial Growth and Biofilm Formation of Multidrug-Resistant Acinetobacter baumannii
Source: Microorganisms. 2025 Mar 11;13(3):639. doi: 10.3390/microorganisms13030639 (PMC11944726; doi:10.3390/microorganisms13030639)
Supplement: Supplementary file 1 [file microorganisms-13-00639-s001.zip › Supplementary Files/Supplementary Figure S1.pdf]

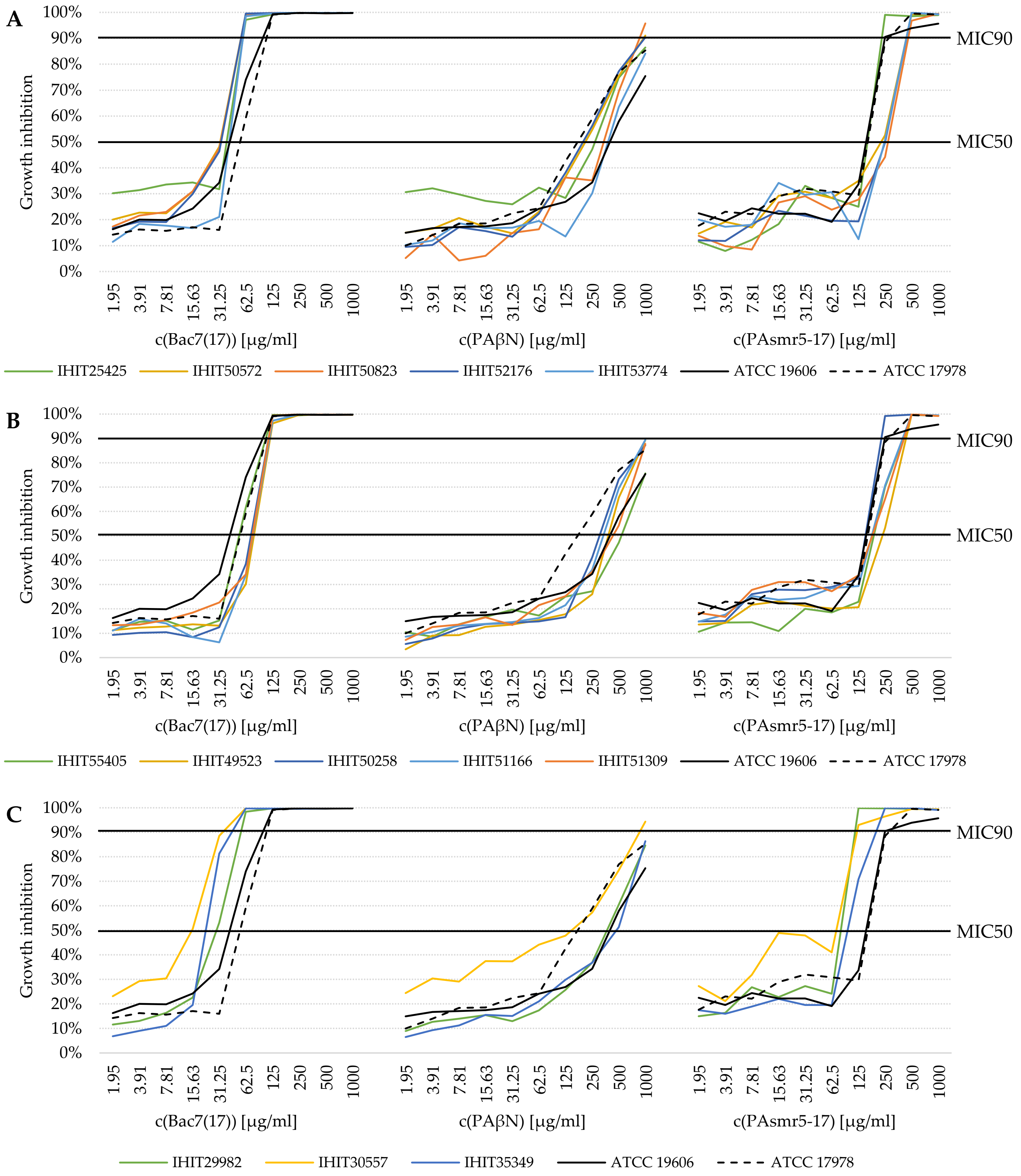

Figure S1. Growth inhibition (%) by the AMPs Bac7(17), PAβN and PAsmr5-17 of 13 MDR-*A. baumannii* isolates belonging to clonal lineages (**A**) IC1 (n = 5 isolates), (**B**) IC2 (n = 5 isolates), (**C**) IC7 (n=3 isolates) and the reference strains ATCC 19606^{T} and ATCC 17978. All assays were carried out in triplicates, and the results were averaged. The MIC describes the lowest concentration at which no bacterial growth can be detected. MIC90: 90% growth inhibition. MIC50: 50% growth inhibition.
